# Standards of health promoting hospitals: 68 or 40 measurable elements?

**DOI:** 10.15171/hpp.2017.20

**Published:** 2017-06-14

**Authors:** Akbar Nikpajouh

**Affiliations:** Rajaei Cardiovascular Medical and Research Center, Iran University of Medical Sciences, Tehran, Iran

## Dear Editor,

Regarding the article “Health promoting hospitals: a study on educational hospitals of Isfahan, Iran” published in the prestigious journal, *Health Promotion Perspectives*, the respected authors of the mentioned article have used 68 measurable elements.^1^ Here while I would like to thank you and the writers of that journal, I deemed it necessary to explain some points to be considered in similar future studies.

To tell the story of how the International Network of Health Promoting Hospitals & Health Services (HPH) came into existence, we have identified six different time periods or phases. In order to do this, we picked up the lead from the previous work, and hopefully made the developments even clearer. The six development phases of HPH, which will be described in the following, were not planned or well defined at the time. No detailed action plans, evaluations or similar content existed. Therefore the phases were reconstructed and are thus more or less distinct, partly over-lapping at times, with different foci of emphasis, specific milestones and etc. We have named the first period “pre-phase” and the others “phases 1 to 5”.^2^ In phase 4: Standardizing the concept & linking it to quality and evidence (2001-2005), the working group on “Standards for Health Promotion in Hospitals” developed and tested 5 more focused standards for health promotion in hospitals”.^3^ The standards of health promoting hospitals, which were published first in 2004 by the World Health Organization (WHO), included 5 standards containing totally 68 measurable elements.^4^ Yet, the first edition was for the pilot health promoting hospitals (Table 1). However, following the pilot, the authors of the WHO reviewed the standards and lessened the number of measurable elements to 40 in total^5^ (Table 2). Since I intended to perform a study regarding health promoting hospitals, I noticed this change in the number of measurable elements. Hence, I communicated with the author of these series and he mentioned that the edited version in 2006 with 40 measurable elements, would be valid until the publication of a new version. Please kindly enlighten this issue in your prestigious journal so that the researchers do not use the old version with 68 measurable elements published in 2004, while citing the article “Health promoting hospitals: a study on educational hospitals of Isfahan, Iran” and instead use the most recent version with 40 measurable elements edited in 2006 in their studies and self-assessments of hospitals, until a new edition will be available.

**Table 1:** Overall assessment of standards compliance (World Health Organization 2004)

		**Measurable element**
Standard 1	Management Policy	17
Standard 2	Patient Assessment	8
Standard 3	Patient Information and Intervention	8
Standard 4	Promoting a Healthy Workplace	16
Standard 5	Continuity and Cooperation	19
		Total = 68

**Table 2:** Overall assessment of standards compliance (World Health Organization 2006)

		**Measurable element**
Standard 1	Management Policy	9
Standard 2	Patient Assessment	7
Standard 3	Patient Information and Intervention	6
Standard 4	Promoting a Healthy Workplace	10
Standard 5	Continuity and Cooperation	8
		Total = 40

## Ethical approval

None to be declared.

## Competing interests

Author declares that he has no competing interests.

## Author’s contribution

AN is the single author of the paper.
